# Dependency of hydration and growth conditions on the mechanical properties of oral biofilms

**DOI:** 10.1038/s41598-021-95701-4

**Published:** 2021-08-10

**Authors:** J. Pattem, M. Davrandi, S. Aguayo, B. Slak, R. Maev, E. Allan, D. Spratt, L. Bozec

**Affiliations:** 1 Division of Biomaterials and Tissue Engineering, UCL Eastman Dental Institute, University College London, London, UK; 2 Division of Microbial Diseases, UCL Eastman Dental Institute, University College London, London, UK; 3 School of Dentistry, Faculty of Medicine, Pontificia Universidad Catolica de Chile, Santiago, Chile; 4 Department of Electrical and Computer Engineering, University of Windsor, Windsor, Canada; 5 Faculty of Dentistry, University of Toronto, Toronto, Canada; 6 National Centre for Molecular Hydrodynamics, and Soft Matter Biomaterials and Bio-Interfaces, University of Nottingham, The Limes Building, Sutton Bonington Campus, Sutton Bonington, Leicestershire, LE12 5RD UK

**Keywords:** Biological techniques, Biophysics, Microbiology

## Abstract

Within the oral cavity, dental biofilms experience dynamic environments, in part due to changes in dietary content, frequency of intake and health conditions. This can impact bacterial diversity and morpho-mechanical properties. While phenotypic properties of oral biofilms are closely related to their composition, these can readily change according to dynamic variations in the growth environment and nutrient availability. Understanding the interlink between phenotypic properties, variable growth conditions, and community characterization is an essential requirement to develop structure–property relationships in oral-biofilms. In this study, the impact of two distinct growth media types with increasing richness on the properties of oral biofilms was assessed through a new combination of *in-vitro* time-lapse biophysical methods with microbiological assays. Oral biofilms grown in the enriched media composition presented a decrease in their pH, an increase in soluble EPS production, and a severe reduction in bacterial diversity. Additionally, enriched media conditions presented an increase in biofilm volumetric changes (upon hydration) as well as a reduction in elastic modulus upon indentation. With hydration time considered a major factor contributing to changes in biofilm mechanical properties, we have shown that it is less associated than media richness. Future investigations can now use this time-lapse approach, with a clearer focus on the extracellular matrix of oral biofilms dictating their morpho-mechanical properties.

## Introduction

Oral biofilm formation on dental surfaces initiates with the attachment of primary colonizers, such as streptococci^1,2^, followed by the attachment of secondary and later colonizers^3,4^. During this process, complex microbial communities are formed and become embedded in a matrix of self-secreted extracellular polymeric substances (EPS)^5–7^. EPS may account for up to 90% of biofilm total mass and is comprised of bound water, polymers such as extracellular DNA (eDNA), polysaccharides, proteins, and lipids of bacterial and salivary origin^8–10^.

The oral cavity supports the growth of a diverse range of bacteria with its relatively stable environmental conditions and stream of nutrients derived from salivary components, gingival crevicular fluid and host diet. Importantly, the host’s diet can provide fermentable carbohydrates such as sucrose which has a strong relationship with caries development^4^. Conversely, frequent consumption of dairy products and high-fibre food has been associated with caries preventative effects^11^. Although the full extent of varying diet on the bacterial and EPS composition of the oral biofilm is not clearly understood, a recent study has shown that the oral biofilm community composition can be modulated by dietary interventions which could have an impact on the EPS constituents^12^.

Within the biofilm, bound water can reach up to 98% of EPS total mass^13^. In a clinical setting, oral biofilms experience dynamic cycles of de- and rehydration due to fluid consumption^14^ and saliva stimulation^15^. Conditions such as Sjögren's syndrome, which present with reduced salivary flow, can affect these further, elevating the risk of developing dental caries^16^. These de- and rehydration cycles, as well as influencing EPS and bacteria directly, will also affect biofilm structure, impacting its mechanical and volumetric properties^17,18^. Monitoring the effect of biofilm hydration on these properties within the oral cavity *in-situ,* still poses significant challenges. Thus, new experimental approaches are needed to apply appropriate techniques allowing the analysis of biofilms in a non-destructive, label-free manner, at a range of short and long-time scales, *in-vitro*. Currently, advanced imaging techniques such as optical coherence tomography (OCT) and atomic force microscopy (AFM) have been employed to study a wide range of biological samples including microbial cells and biofilms^19–25^. OCT provides a depth-resolved analysis of backscattered light, resolving image depths of several millimetres with a lateral and axial pixel resolution of < 5 µm^26^. AFM provides high-resolution information on morphology and mechanical properties of bacterial cells and EPS^27–30^. Recent attempts to monitor biofilm biophysical changes include using a combination of OCT and AFM to identify changes in mesoscale morphology and nanomechanical properties of biofilms, as a result of age and nutrient availability^31^. Although this previous research highlighted the need for a careful understanding of biofilm ultrastructure when assessing its physical properties, it did not consider the dynamic biofilm hydration, nor its impact upon the biofilm structural and mechanical properties.

In the *in-vitro* setting, investigators often apply a method termed *physisorption* to specimens before analysis. This is the process of air-drying biological tissues onto desired substrates^31,32^. Physisorption enhances specimen surface attachment and reduces movement or drifting during non-destructive studies^33^. During physisorption, i.e., de- and rehydration, biofilms exhibit changes in water content, significantly impact their structural and mechanical properties^13,32^. This is evident in the literature, as investigators have shown significant variations in Young's modulus between biofilms under dry and fully hydrated conditions^27,28,30,34^.

This study aims to investigate how two distinct types of growth media with increasing richness used to culture oral biofilms can affect both their microbiological and biophysical properties. Additionally, this study brings the first evidence on the impact of physisorption to biofilms upon rehydration. Finally, this study seeks to determine whether hydration time is a dependent or independent variable on the structural and mechanical properties of oral biofilms *in-vitro.* In addition, it seeks to understand how growth media richness can influence bacterial diversity and EPS composition. Finally this study also seeks to determine the effect of sample preparation and storage conditions on the mechanical properties of healthy human plaque.

## Materials and methods

### Hydroxyapatite substrates

Twenty-four 5 mm diameter, 2 mm height HAP disks (Clarkson Chromatography Products, USA) were polished using P4000 SiC grit paper (Sigma-Aldrich) and randomly assigned into 2 groups of 12 for biofilm production.

### Biofilm production

Biofilms were formed from pooled human saliva (PS), N = 23 healthy volunteers [self-reported] aged between 20 and 60 using two distinct types of growth media. Ethical approval for the analysis of pooled saliva as part of this project were obtained from University College London (UCL) Ethics Committee (project number 5017/001). The ethics committee approved the consent procedures for the sample collection and processing. All national, regional, and institutional guidelines provided by the ethics committee were adhered to with written informed consent requested and collected from each participant before providing saliva samples. Participants were assured that their data was treated confidentially, anonymously, and informed that their data may be used for the purpose of publication. This was sought from the UCL Data Protection Officer and the departments Data Protection Co-ordinator.

Firstly, a basic (low carbon, denoted LC) and secondly, an enriched (high carbon, denoted HC) growth medium. The basic growth medium (LC) consisted of artificial saliva, containing 1 g/L of lab-lemco, 2 g/L of yeast extract, 5 g/L of protease peptone, 2.5 g/L of mucin from porcine stomach (Type III), 0.35 g/L of sodium chloride, 0.2 g/L of calcium chloride and 0.2 g/L potassium chloride, 0.05% (w/v) urea and 0.1% (w/v) sucrose. The enriched growth medium (HC) contained an additional 50% (w/v) brain heart infusion and 5% sucrose (w/v). Microcosm biofilms were grown directly on sterilized HAP discs using a feed batch culture approach in a 96-well plate (Nunc Nunclon Delta). All materials were sourced from Sigma-Aldrich, UK. Biofilms cultured using both growth conditions were then randomly assigned into two subgroups for biophysical and microbiological analysis. The inoculum was prepared by mixing PS and growth medium at 1:7 ratio, and 180 µL aliquots (~ 4.5 × 10^6^ CFU) of the inoculum were added into wells and incubated at 37 °C in air containing 5% CO_2_. Growth medium was replaced at 24 h intervals, and biofilms were harvested at the end of the incubation period.

## Microbiological analysis

### Determination of viable counts and pH

Biofilm-coated HAP discs were removed and rinsed twice by gently dipping into sterile phosphate-buffered saline (PBS). Specimens were then transferred into 1.5 mL screw-cap tubes containing 1 mL PBS and 5 large glass beads (2.5–3.5 mm diameter). Biofilms were then disrupted by vortexing for 45 s, and further homogenized in a sonicating water bath at 35 kHz (30 W) for 10 min, at room temperature. An aliquot of 200 µL was removed and serially diluted tenfold in pre-reduced PBS. A 20 µL aliquot of each dilution was spread and plated on Columbia blood agar (CBA) (Sigma-Aldrich, UK), and fastidious anaerobe agar (FAA) (Sigma-Aldrich, UK) supplemented with 5% (v/v) defibrinated sterile horse blood. CBA plates were incubated in air containing 5% CO_2_ at 37 °C and FAA plates in an anaerobic chamber (80% N_2_, 10% H_2,_ and 10% CO_2_) at 37 °C, to obtain total aerobe and total anaerobe counts, respectively.

The pH of the spent medium was measured at the end of incubation (day 5), using a Jenco 6230 N portable pH meter combined with an InLab nano glass electrode (Mettler Toledo). The group-wise comparison was performed using Mann–Whitney U test.

### Water-soluble carbohydrate and protein content analysis of EPS

Homogenized biofilm suspensions were centrifuged (10,000×*g*) for 5 min, and the supernatants were retained. Pellets were washed with 600 µL PBS and centrifugation was repeated. For each sample, supernatants (2x) were pooled and filtered through 0.22 µm filters and used for the determination of water-soluble carbohydrates and total protein. Water-soluble carbohydrates were determined by an anthrone-sulphuric acid assay using glucose as a standard. Quantification of total protein in EPS was performed by using a Pierce BCA Protein Assay (Sigma-Aldrich, UK) using bovine serum albumin (BSA) (Sigma-Aldrich, UK) as a standard. The level of statistical significance was calculated by the Mann–Whitney U test.

### Biofilm community characterization

Total genomic DNA was extracted from pooled saliva and biofilm samples using PowerSoil DNA Isolation kit (Qiagen, UK). Extractions were performed with the alternative digestion method provided in the manufacturer's protocol in which samples were incubated at 70 °C for 10 min in lysis solution before the bead beating step. Finally, DNA was eluted in 75 µL molecular grade water, quantified on a NanoDrop spectrophotometer (Thermo Scientific, UK) and stored at − 20 °C. The V3–V4 hypervariable region of 16S rRNA gene was amplified by PCR from approximately 25 ng metagenomic DNA using universal 341F (5`-CCTACGGGNGGCWGCAG-3`) and 805R (5`-GACTACHVGGGTATCTAATCC-3`) primers fused with Nextera XT index and MiSeq adapter sequences. The library was generated by pooling equimolar concentrations of cleaned amplicons and quantified by Qubit assay (Thermo Scientific, UK). Subsequently, 4 pM library containing 10% 12 pM PhiX was loaded according to Illumina's instructions (January 2016) for MiSeq 2 × 250 bp paired-end sequencing run (Illumina, v2 kit). Sequencing data were analyzed using 'Quantitative insights into microbial ecology 2' (QIIME2 version 2019.7, https://qiime2.org/). The raw sequences were de-multiplexed, and de-noised using the DADA2 algorithm with default parameters to create amplicon sequence variants (ASVs). The mean sequencing depth was 87,104 (range of 41,999–115,558), and the resulting ASVs were assigned taxonomy using the Human Oral Microbiome Database 16S rRNA gene reference sequence (HOMD v14.5) (37). Alpha diversity was calculated on ASVs using the observed ASV index and Shannon index. Genus level taxonomic profiles were generated at ≥ 1% relative abundance threshold.

## Biophysical analysis

### Optical coherence tomography

A VivoSight Multi-Beam Swept Source OCT system (Michelson Diagnostics Ltd, UK) was used to observe the cross-sectional and en-face morphology of each biofilm, by employing a class I laser (λ = 1305 nm) and a scan rate of 10 kHz. The default scanning volume was set to 6 mm × 6 mm and approximately 2 mm deep. Specimens were attached to a 35 mm petri dish (Thermofisher, UK) using perfluoropolyether lubricant (Fomblin, UK). Imaging was conducted dry at baseline, and after PBS hydration, images were collected every 2 min for a total time of 100 min. For each sample, a total of 500 B-scans were recorded. Each B-scan was recorded 10 µm apart at a pixel size of 4.53 µm. A custom in-house MATLAB algorithm (The MathWorks, Inc., Natick, MA, USA) was employed to determine the volumetric change (mm^3^) of each biofilm by profiling individual OCT A-scans. The reference scan was taken at 2 min PBS exposure with further measurement scans consequently at 2 min intervals up to 100 min. The algorithm extracted surface topography and specimen substrate within every B-scan by applying a digital band-pass filter, signal envelope, and local peak detection algorithm on each, respectively. A series of 1-way ANOVAs (p < 0.05) were used to determine statistical differences between LC and HC normalized volumetric changes and consecutive 20 min intervals within groups.

### AFM probe modification and time-lapse force spectroscopy

A JPK Nanowizard AFM (Bruker Nano, Santa Barbara, CA) was used to modify NPO-10 tip-less cantilevers (Bruker Ltd, France). Cantilevers were modified with 50 µm borosilicate spheres (Whitehouse Scientific, UK) using UV-curing resin (Loctite, UK). Successfully modified cantilevers were calibrated before analysis generating a spring constant of 0.3 ± 0.05 N/m. Operating in force-spectroscopy mode, the AFM was used to monitor one area of each biofilm specimen at baseline (n = 150). After rehydration with PBS, the specimen area was monitored continuously for 100 min with a retract delay of 5 s and a total 10 s for each force-distance curve, for a total of 18 force-distance curves per minute per group. Young's modulus (kPa) values were obtained from individual force-distance curves, using proprietary software (JPK Data Processing Software, Version 4.2, https://www.nanophys.kth.se/nanolab/afm/jpk/manuf-manuals/DPmanual.4.2.pdf, Bruker Nano, Santa Barbara, CA). Young's modulus of specimens was extracted using the Hertzian model for a spherical indenter^21,30^. Young's Modulus data were graphed at 5 min intervals (n = 90 per time point), and statistics were performed at 20 min intervals (n = 360) using a series of 2-way ANOVAs (p < 0.05) between groups and exposure times. All experiments were performed in triplicate, for both HC and LC groups, including corresponding microbiological, volumetric, and mechanical analysis.

## Results and discussion

The experimental protocol used in this study to grow dental plaque biofilms was designed to provide fresh nutrients daily, and to generate divergent biofilms using two distinct growth medium formulations. Direct sampling of human dental plaque was considered but ruled out because of the likely disruption in the biophysical properties of the biofilm during recovery. Therefore, an *in-vitro* approach was considered favourable especially since the use of human saliva from volunteers has been successfully used to generate microcosm biofilms *in-vitro*^35,36^. Though, it must be noted that there is continuing growth medium developments with many modifications for growing oral biofilms to optimise growth while preserving the native diversity^37–39^. In this study, artificial saliva was chosen as the base medium and was enriched with brain heart infusion and sucrose supplementation to mimic the diversity in host diet^11,12^. Another crucial aspect was the maturity of the oral biofilms. However, ‘maturity’ for oral biofilm is still a loosely defined term, and essentially refers to compositional maturity on the basis of biofilm age, which could be in the scale of hours or days^40–43^.

### Total viable counts and biofilm pH

**Figure 1:**
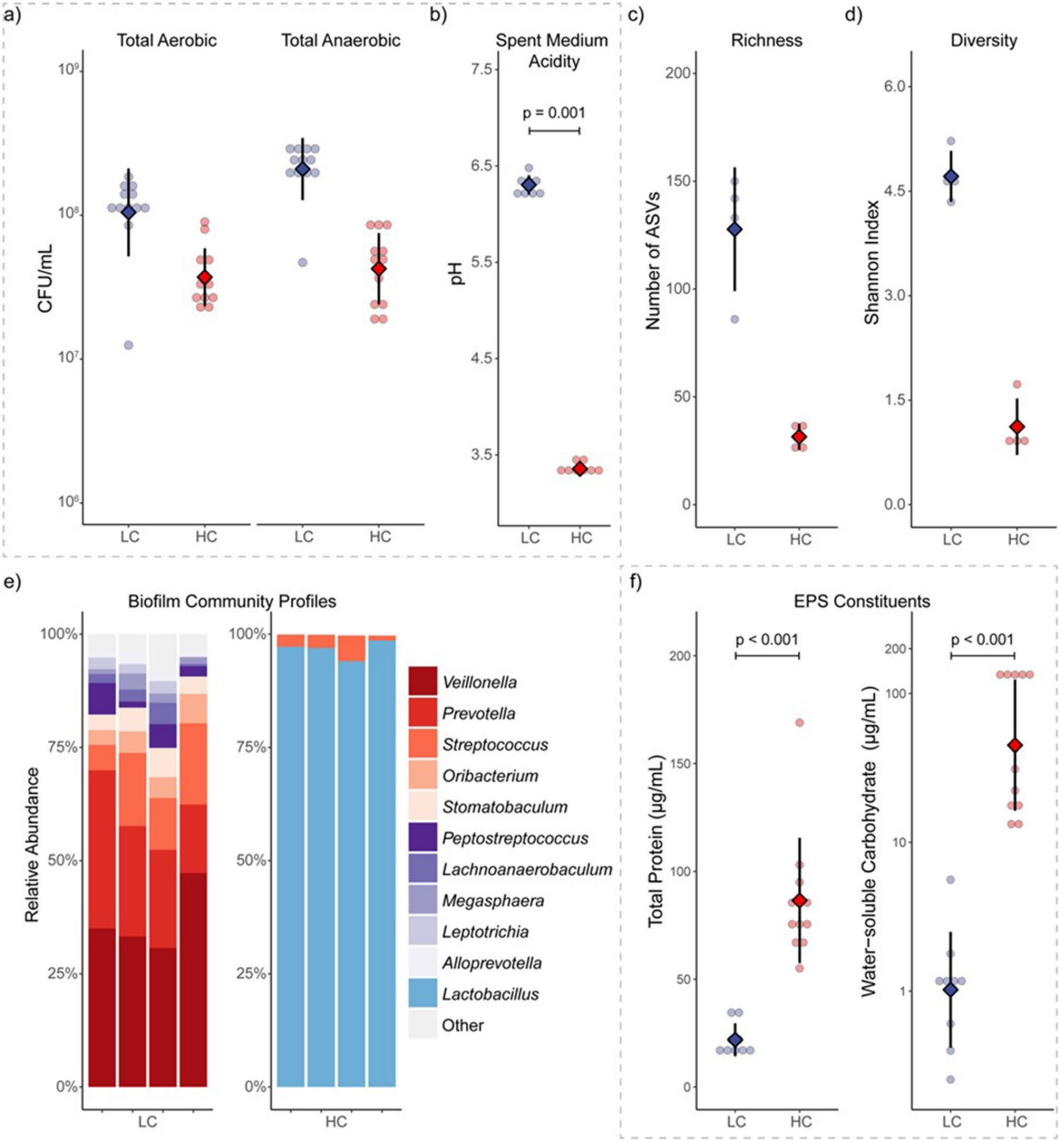
Characterisation of the biofilms grown using basic (LC, blue) or enriched (HC, red) growth medium diet (n = 3). (**a**) Showing the number of total viable bacteria after 5 days of incubation in 5% CO_2_ air, and (**b**) spent medium pH of the LC and HC groups. (**c**) Community richness and (**d**) diversity of the respective biofilms, and (**e**) genus level taxonomic profiles showing genera with ≥ 1% relative abundance. (**f**) EPS composition of the LC and HC biofilms as measured by total protein and water-soluble carbohydrate. Between group comparisons were performed using Mann–Whitney U test.

Based on our preliminary research using the protocol reported in this study, it was found that the biofilm growth was stable after day 3 with no further increase in viable counts (data not shown). For the current study, with an additional 2 days of incubation after which the steady-state biofilms were established, the biofilms on day 5 were assumed to be stationary. At that point, the LC group had viable bacterial counts reaching 8.2 log_10_CFU while slightly lower counts at 7.6 log_10_CFU in HC biofilms (Fig. 1a). In terms of total aerobic and anaerobic bacteria, LC grown biofilms contained increased number of anaerobic bacteria compared to the HC grown biofilms, which could be due to increased community diversity that created oxygen depleted micro-habitats within the biofilm structure^44^. In terms of environmental acidity, the pH of the spent medium was significantly lower for HC (pH 3.4) compared to LC groups (pH 6.4) at the end of the incubation period (Fig. 1b).

The impact of the high and low nutrients was further revealed by community-level analysis using 16S rRNA gene sequencing data. LC groups, which closely mimic nutrient-poor human saliva, supported biofilms with richness and diversity comparable to *in-vivo* plaque communities (Fig. 1c,d)^44–47^. Presumably, one of the factors that facilitated the development of such biofilm community was bacterial cooperation and complementation. For example, mucin could provide nutrients with its carbohydrate moieties as well as protein backbone. However, complementary synergistic interactions are necessary for its complete utilisation which could be a factor enabling increased diversity among the biofilm community (Bradshaw et al. 1994). Moreover, metabolic by-products could be an important driver for diversity by providing additional nutrients that were initially not available in the growth medium such as lactate which can be metabolised by *Veillonella* species ^48^. The breakdown of organic acids (e.g. lactate) is also important for maintaining a neutral pH which was evident in LC group ^49^*.*

In contrast, the community richness (number of ASVs) in HC biofilms was in the range of 24–37, and the greatly reduced Shannon diversity index (1.1) was indicative of a highly imbalanced biofilm community^50,51^. Given the highly acidic spent medium pH, expectedly, genus level taxonomic profiles showed dominance of aciduric and acidogenic lactobacilli for HC grown biofilms (Fig. 1e)^52^, characteristic of a high sucrose diet induced cariogenic biofilm^4,53^. Such a low pH may also induce stress on the micro-consortia, consequently affecting the mass, morphology and mechanical properties of the formed biofilms. This may also hydrolyse some biofilm components such as those found in EPS^54,55^. While artificial saliva added to the growth media was unable to buffer such a low pH in HC biofilms, future studies should test a gradient of sucrose concentration and BHI to demonstrate a clearer correlation between sucrose concentration, pH and its effect on resulting microbiological and physical properties.

Overall, the community characteristics of the HC biofilms poorly reflected the complexity of *in-vivo* biofilms^45^. The spent medium pH and community diversity metrics were suggestive of an oral biofilm similar to an *in-vivo* carious biofilm. Provided that the aim of this protocol was to generate divergent oral biofilms to study the effect of EPS composition, the resultant biofilms were sufficient for the current study.

### Total protein and water-soluble carbohydrate analysis

As shown in Fig. 1f, the total protein and water-soluble carbohydrate constituents of EPS were highly influenced by bacterial community composition. Both total protein and water-soluble carbohydrates were found to be significantly greater in HC group compared to the LC group biofilms (p < 0.003). Total protein and water-soluble carbohydrate content in HC groups were 86.5 µg/mL and 68.8 µg/mL, compared to 21.9 µg/mL and 1.5 µg/mL for LC groups. In biofilms, EPS serves fundamental physical and biological functions such as enhancing biofilm adhesion to dental surfaces, mediating co-adhesion of bacteria, and providing nutrients during starvation^6,10^. Additionally, EPS has an essential role in maintaining mechanical stability against shear forces^6,56,57^. With that, we next aimed to study the role of hydration in such morphological and mechanical properties by using these two distinct biofilms with differing community and EPS characteristics.

Sonication, particularly the use of direct pulsing sonication is typically used to disperse biofilms^58^. In our study, we applied vortexing and subsequent sonication to our HAP-biofilms in an Eppendorf via the use of a sonication water bath. Vortexing alone showed high viable cell recovery from biofilms, however, resulted in overlapping colony formations on agar plates (data not shown). The introduction of sonication enabled us to destabilize the biofilms more efficiently and break down the aggregated cell clumps allowing us to effectively monitor bacterial counts and soluble EPS components. We applied this vortexing and sonication method across all analysed biofilms to enable effective group comparison. In future, we will apply the microscopy for validation, enabling us to confirm the effect of sonication on acquired biofilm bacterial counts and EPS analysis. Including, the use of direct pulse sonication for comparison.

### Biofilm rehydration volume analysis

**Figure 2:**
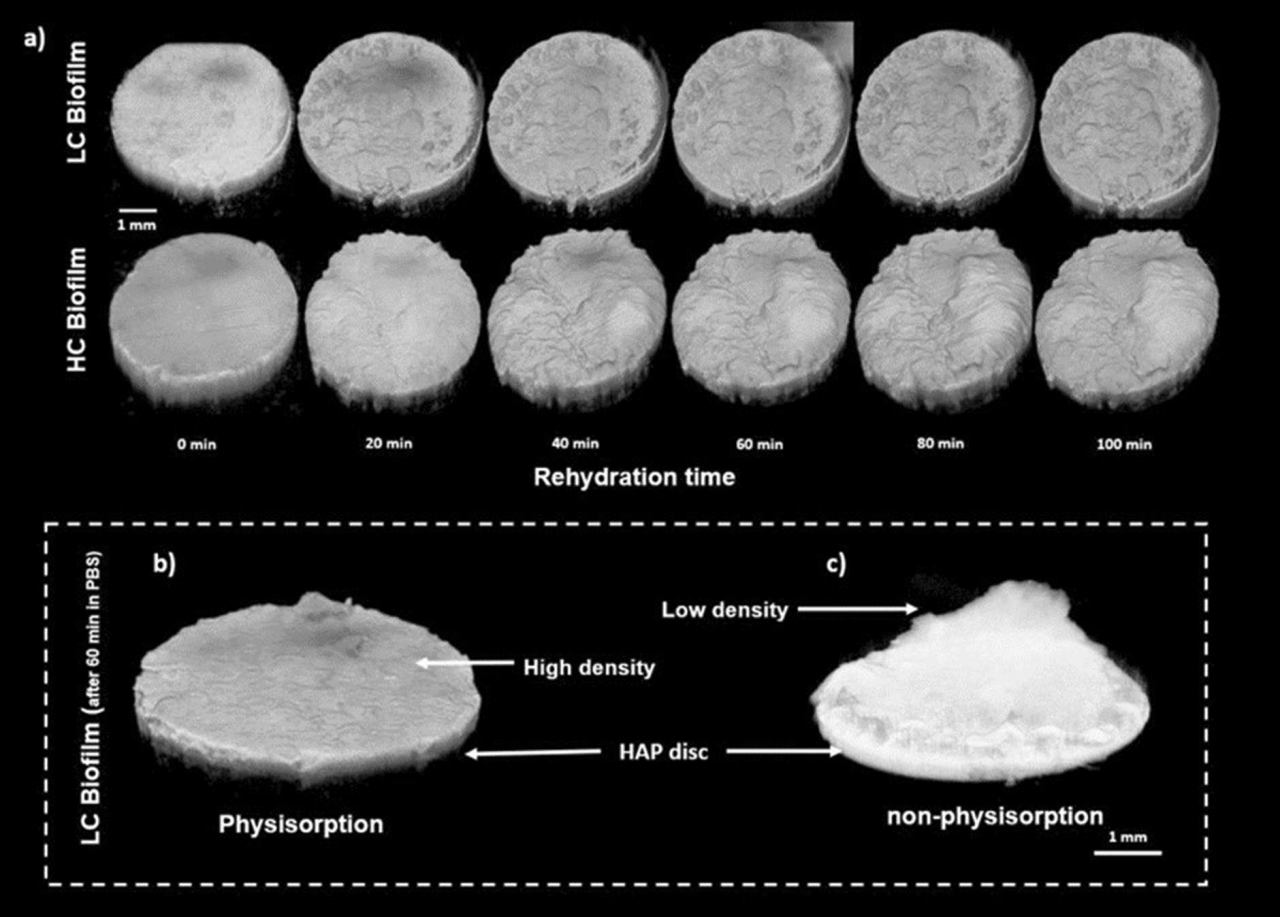
Showing (**a**) OCT images of both LC and HC biofilms at 20 min intervals up to 100 min, (**b**) showing effect of physisorption on LC biofilms after 1 h physisorption with (**c**) showing non-physiosorbed under PBS hydrated conditions.

Figure 2a shows example 3D OCT images of LC and HC biofilms respectively, at baseline (dry) and 20 min intervals up to 100 min. The use of 1-h physisorption provided higher density biofilms on the HAP disc surface shown in Fig. 2b compared to non-physisorped biofilms shown in Fig. 2c. This enabled effective characterisation of volumetric and mechanical property changes across the hydration regime, due to a clear distinction between the biofilm surface and surrounding PBS.

**Figure 3:**
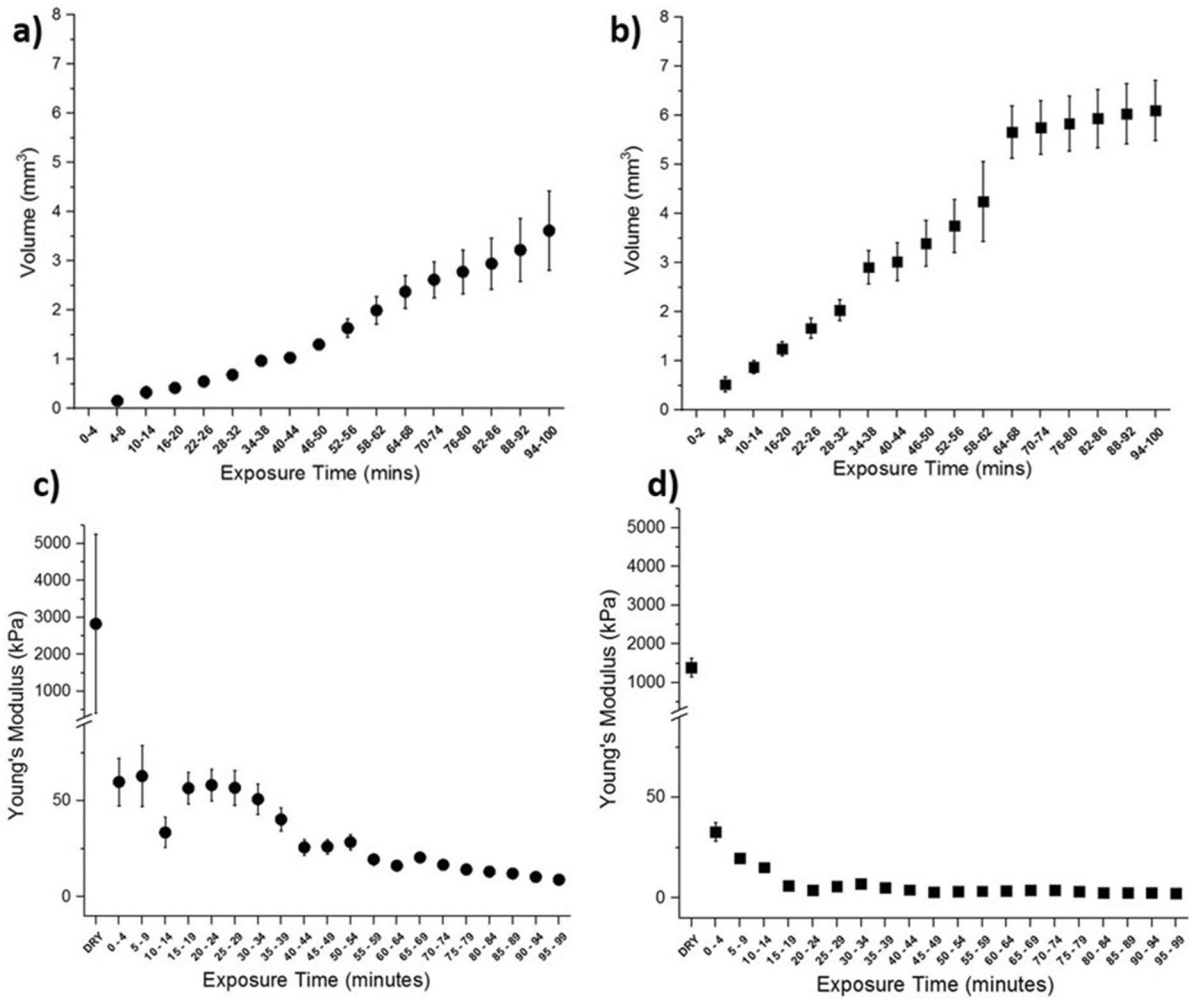
Showing (**a**) and (**b**) the volumetric changes of oral biofilms with LC and HC contents respectively. (**c**) and (**d**) The Youngs Modulus of LC and HC biofilms respectively at 5 min intervals.

Overall, LC and HC biofilm volume increased over the hydration time. However, there was no statistical difference in the normalized volume change between LC and HC biofilms at each 20 min interval (p > 0.05). Within groups, both LC and HC biofilms exhibited significant changes in volume only after 60 min (p < 0.05), with no subsequent significant differences when continuing to hydrate (p > 0.05), shown in Fig. 3a,b. Figure 2b,c show examples of a rehydrated LC biofilm with or without prior physisorption. Both these images highlight morphological differences in the biofilm's architecture because of the physisorption/rehydration process. Physisorption induces a collapse of the biofilm ultra-structure through the drying process, resulting from a reduction in bound water content^13^. This collapse of biofilm structure increases the contact area between HAP and the EPS/biofilm, enabling a more secure attachment to the surface. It is also clear the biofilm does not recapitulate its original swollen structural appearance once rehydrated, suggesting architectural plastic deformation of the biofilm has occurred.

## Biofilm rehydration mechanical properties: Young’s modulus (E_s_)

As previously demonstrated, physisorption can induce morphological changes in the overall structure of the biofilms shown in Fig. 2b,c. This structural alteration is likely to be accompanied by a change in its mechanical properties. To investigate this, AFM-based indentations were performed directly on physisorbed biofilms using a functionalized probe as described elsewhere^23,31^. LC biofilms were found to be significantly stiffer compared to HC biofilms after 1 h physisorption (p < 0.05), exhibiting values of Young's Moduli E_s_ 2.83 ± 1.22 MPa and 1.39 ± 0.12 MPa, respectively. Young's moduli values corroborated previously reported data on single-species biofilms after 1 h thermal drying, using similar indentation methods^21,27,30,34^. After 100 min rehydration, a similar trend in Young’s modulus was also found, with LC and HC biofilms exhibiting E_s_ = 10.4 ± 6.4 kPa and 2.8 ± 2.1 kPa, respectively. Again, coinciding with previously reported ranges^21,27,30,34^. As expected, HC biofilms presented a lower value for Young's modulus in rehydrated conditions owing to its higher EPS content^31^. However, since significant changes in the structure of the biofilms were recorded because of the rehydration, it is likely that the elastic properties would fluctuate until a rehydration steady state is achieved. This progression towards biofilm a steady state was assessed by OCT volumetric analysis as presented in Fig. 3a,b. LC biofilm volume increased steadily over the 100 min rehydration protocol, whereas the HC biofilm reached a plateau after 1 h. Figure 3c,d show E_s_ values, plotted at 5 min intervals of LC and HC biofilms respectively, after physisorption over 100 min. Both LC and HC biofilms exhibited a large reduction in E_s_ in the first 5 min of rehydration, with a significant reduction after 20 min (p < 0.05). Throughout the rehydration, LC biofilms showed higher E_s_ at each 5 min interval and were significantly greater at each 20 min interval, compared to HC biofilms (p < 0.05). At 5 min intervals from 80 to 99 min, all LC E_s_ values were also consecutively lower (p < 0.05), indicating a further change in Youngs modulus shown in Fig. 3c, even after 100 min (data not shown). At 5 min intervals from 80 to 99 min, all HC E_s_ values were not significantly different at each time increment (p > 0.05). Therefore, mechanical stabilization was found to be much earlier for HC compared to LC biofilms, with no meaningful change in HC biofilm E_s_ after 20–39 min (p > 0.05) shown in Fig. 3d.

Biofilms grown with enriched medium (HC), where EPS are most prevalent, the elasticity of the biofilm reached a plateau much sooner than in the case of low EPS content biofilms (LC grown). Similarly, the volume of the biofilm seems to be stabilizing sooner in the case of HC when compared to LC.

As EPS are known to be the principal determinant of biofilm's mechanical properties, it is hypothesized that the increased production of soluble EPS significantly reduced the biofilm E_s_ in both dry and full hydrated states^31,59,60^. Since EPS are the main body for bound water content, an increase in EPS content may increase biofilm osmotic potential^60–63^. As the hydration regime progressed, it was predicted an increase in composite biofilm bound, and free water content ensued, resulting in a decrease in E_s_. Therefore, we can postulate that soluble EPS content, rather than hydration time is more associated with the mechanical stabilization of biofilms grown in either basic or enriched culture media. To further evaluate the role of soluble and insoluble EPS components on the microbiological and physical properties of biofilms, future investigators may wish to perform analysis over a gradient of BHI and sucrose concentrations. Multi-linear regression analysis can be applied using total EPS mass (soluble and insoluble) and rehydration time as independent variable with physical characterisation as dependent variables.

This study highlights the effect of variations in sucrose on dental biofilm formation and their corresponding microbiological and morpho-mechanical properties. Particularly, the effect of increasing sucrose concentration on reducing biofilm bacterial diversity, enhancing its cariogenic potential via a reduction in pH. The observations of this study provide practicality and clinical utility in oral health, such as providing clinical advice for dental practitioners to pass on to patients about maintaining a good oral microbiome and practicing good oral health.

## Conclusions

This study sought to determine the effect of growth media richness on the community structure of oral biofilms upon rehydration. Additionally, their effect on the morphological and mechanical properties as a function of hydration time was also determined. Using a multiscale, continuous monitoring approach, it was demonstrated that using a rich-growth medium led to a decrease in pH, consequently decreasing bacterial diversity and, through a possible stress-response, increased soluble EPS production. Therefore, the increase in soluble EPS may not solely depend on culture richness, but also the pH-induced response caused by a severe reduction in bacterial diversity. Consequently, this increased the oral biofilm volumetric properties while reducing Young's modulus, reflected over the entire hydration regime. Increasing soluble EPS production increased the potential for bound water content and is postulated to positively impact the stability or plateaux in biophysical properties of oral biofilms. To date, this is the first study of its kind combining microbiological and multiscale techniques for time-lapse monitoring the rehydration process of oral biofilms, *in-vitro*. It is believed this approach can be used to monitor the effect of other variables on the microbiological and morpho-mechanical properties of oral biofilms, such as pH, salt concentration, soluble and non-soluble EPS components and osmotic potential.
